# Recombinant Baculovirus-Produced Grass Carp Reovirus Virus-Like Particles as Vaccine Candidate That Provides Protective Immunity against GCRV Genotype II Infection in Grass Carp

**DOI:** 10.3390/vaccines9010053

**Published:** 2021-01-14

**Authors:** Ting Gao, Caixia Gao, Siyu Wu, Yingying Wang, Jiyuan Yin, Yingying Li, Weiwei Zeng, Sven M. Bergmann, Qing Wang

**Affiliations:** 1 Key Lab of Fishery Drug Creation of Ministry of Agriculture and Rural Areas, Key Lab of Aquatic Animal Immune Technology of Guangdong Province, Pearl River Fisheries Research Institute, Chinese Academy of Fishery Science, Guangzhou 510380, China; gaoting@prfri.ac.cn (T.G.); gaocaixia@prfri.ac.cn (C.G.); wusiyu@prfri.ac.cn (S.W.); eagles5257@prfri.ac.cn (Y.W.); yinjiyuan@prfri.ac.cn (J.Y.); liyingying@prfri.ac.cn (Y.L.); 2 Department of Aquatecture, Tianjin Agricultural University, Tianjin 300384, China; 3 Guangdong Provincial Key Laboratory of Animal Molecular Design and Precise Breeding, Key Laboratory of Animal Molecular Design and Precise Breeding of Guangdong Higher Education Institutes, School of Life Science and Engineering, Foshan University, Foshan 440605, China; davy988@prfri.ac.cn; 4 Institute of Infectiology, Friedrich-Loeffler-Institute (FLI), Federal Research Institute for Animal Health, 17493 Greifswald–Insel Riems, Germany; Sven.Bergmann@fli.de

**Keywords:** grass carp reovirus (GCRV), genotype II, virus-like particles, vaccine, grass carp

## Abstract

Grass carp reovirus (GCRV) leads to severe hemorrhagic disease in grass carp (*Ctenopharyngodon idella*) and causes economic losses in grass carp aquaculture. Recent epidemiological investigations showed that GCRV genotype II is the dominant subtype in China. Therefore, it is very important to develop a novel vaccine for preventing diseases caused by GCRV genotype II. In this study, we employed a bac-to-bac expression system to generate GCRV-II-based virus-like particles (VLPs). Previous studies have shown that the structural proteins VP3, VP4, and VP38 encoded by the segments S3, S6, and S10 of type II GCRV are immunogenic. Hence, the GCRV-VLPs were produced by co-infection of sf9 cells with recombinant baculoviruses PFBH-VP3, PFBH-VP4, and PFBH-VP38. The expressions of VP3, VP4, and VP38 proteins in GCRV-VLPs were tested by IFA and Western blot analysis. By electron microscopic observations of ultrathin sections, purified VLPs showed that the expressed proteins are similar in shape to GCRV genotype II with a size range from 40 nm to 60 nm. The immunogenicity of GCRV-VLPs was evaluated by the injection immunization of grass carp. The analysis of serum-specific IgM antibody showed that grass carp immunized with GCRV-VLPs produced GCRV-specific antibodies. Furthermore, injection with GCRV-VLPs increased the expressions of immune-related genes (IgM, IFN, TLR3, TLR7) in the spleen and kidney. In addition, grass carp immunized with a GCRV-VLPs-based vaccine showed a relative percent survival rate (RPS) of 83.33% after challenge. The data in this study showed that GCRV-VLPs demonstrated an excellent immunogenicity and represent a promising approach for vaccine development against GCRV genotype II infection.

## 1. Introduction

Grass carp (*Ctenopharyngodon idella*) is one of the most important aquaculture species in China. It has been widely cultivated over the last 60 years and has a great commercial value [1]. According to the China Fishery Statistical Yearbook, the species production exceeded 5.5 million tons and accounted for 21.72% of total freshwater fish production in 2019. However, grass carp hemorrhagic disease (GCHD) caused by grass carp reovirus (GCRV) is a serious infectious disease that mainly affects fingerlings during rearing and leads to severe economic losses to aquaculture in China [2]. GCRV belongs to the genus *Aquareovirus* of the family Reoviridae and was the first viral pathogen identified from aquatic animals in China in 1983 [3]. GCRV is a double-stranded RNA (dsRNA) virus, and its genome consists of 11 segments. The virion is a non-enveloped, icosahedral particle with a diameter between 65 and 72 nm and surrounded by multiple concentric capsid proteins [4]. It is known that GCRV strains can be divided into three distinct genotypes (I, II, and III) based on sequence comparison and analysis. The representative isolates are GCRV-873 (I), GCRV HZ08 (II), and GCRV104 (III), respectively [5,6]. Recent epidemiological investigations showed that most GCRV strains isolated are GCRV genotype II in China, which is more virulent and causes severe hemorrhagic symptoms with mortality reaching 80% [5,7]. Therefore, it is very important to develop a novel vaccine for preventing the diseases caused by GCRV genotype II.

Currently, there is no specific treatment against GCHD, and the only available protection has been vaccination strategies. The vaccines for the control of GCHD are inactivated vaccines, attenuated vaccines, subunit vaccines, and DNA vaccines [8,9,10,11]. An attenuated vaccine is the only licensed GCRV vaccine available in the market, which was developed by attenuating the GCRV-892 strain through serial passages in cell culture [10]. However, the disadvantage of using attenuated vaccines is the potential risk of reversion to virulence [12]. The major disadvantage of inactivated vaccines is the limited duration of immunity protection [13]. Moreover, subunit vaccines and DNA vaccines are expensive and time consuming to manufacture, and the safety of DNA vaccines needs to be evaluated [14]. Therefore, it would be highly beneficial to grass carp aquaculture to develop a novel vaccine that reduces the disadvantages of existing vaccines.

Virus-like particles (VLPs) serve as an ideal platform overcoming the limitations of traditional vaccines. The generation of VLPs was first described with the reconstitution of the tobacco mosaic virus (TMV) in 1955 [15]. Since then, VLPs have become unique entities in the vaccine development strategies [16]. The VLPs self-assemble structures by one or several viral structural proteins; therefore the risk of a viral replication can be fully avoided [17]. Additionally, they allow the display of complex and native antigens in a highly repetitive form on their surface, which also can induce both cellular and humoral immune responses [18]. There are five major protein expression systems that can be constructed by VLPs. The bac-to-bac baculovirus expression system is one of them, which has advantages of post-translation modification, high biological activity, and efficient expression of foreign genes [19,20]. Furthermore, the baculovirus cannot infect mammals; therefore, it is not a safety concern to humans [21]. In light of these advantages, a bac-to-bac expression system was employed to generate GCRV genotype II based VLPs.

Previous studies have shown that the structural proteins VP3, VP4, and VP38 encoded by the segments S3, S6, and S10 of GCRV genotype II have a good immunogenicity. The helicase VP3 is encoded by the segment S3, which is involved in the transcription and capping of viral RNAs [22]. The main outer capsid protein VP4 is encoded by the segment S6 and has been reported to be involved in viral infection and replication [23]. The segment S10 encodes the structural protein VP38, which can generate neutralizing antibodies [24]. Moreover, these proteins have been investigated to serve as the potential vaccine against GCRV. Immunization of grass carp with a recombinant VP4 protein exhibited immune-protective effects, and the specific IgM levels were significantly up-regulated [25]. A subunit vaccine based on outer capsid proteins VP4 and VP35 of GCRV type II has been shown to induce immunity and exhibited 67% survival. Chen et al. (2018) vaccinated grass carp with a DNA vaccine consisting of the VP4, and the relative percentage of survival (RPS) was about 59.9% after the grass carp were challenged with GCRV [26,27]. VP38 can induce high titers of specific antibodies [28]. Therefore, these proteins encoded by those segments are good candidates to design GCRV vaccines.

In this study, we developed VLPs containing VP3, VP4, and VP38 structural proteins of the HuNan1307 strain of GCRV genotype II, which was employed as the VLP -based vaccine to immunize grass carp. Then, the immunogenicity and protection ability of the GCRV VLP-based vaccine were evaluated against a virus challenge. The data showed that the GCRV VLP-based vaccine could provide protection to grass carp and represents a novel approach for vaccine development against GCRV genotype II infection.

## 2. Materials and Methods

### 2.1. Virus and Cells

GCRV-HuNan1307 used in this study was isolated from infected grass carp in a fish farm located in Zhuzhou (Hunan, China). The complete genome sequences of HuNan1307 are publicly available (GenBank accession number: S1–S11, KU254566-KU254576). Insect cells (sf9) were cultured at 28 °C in Sf9-900 II complete medium (Gibco, Gaithersburg, MD, USA).

### 2.2. Construction of Recombinant Baculoviruses

GCRV genomic dsRNA was extracted using a Trizol Reagent kit (Invitrogen, Carlsbad, CA, USA) according to the protocol of manufacture. Afterward, reverse transcription was performed to produce cDNA, which was used to clone the segments S3, S6, and S10 of the isolate GCRV-HuNan1307 (GenBank accession number KU254568, KU254571, KU254575). The target genes were amplified by PCR using primers with appropriate restriction enzymes according to the multiple cloning sites of the donor plasmid, pFastBac^TM^ HTA (MiaoLing, China) (Table 1, the underlined indicate nucleotide restriction sites), and amplification was performed using high-fidelity enzyme PrimeSTAR GXL DNA polymerase (TaKaRa, Beijing, China). After purification and recycling using the E.Z.N.A.^TM^gel extraction kit (Omega, Beijing, China), the PCR products were ligated to the plasmid pMD18-T (TaKaRa, Beijing, China), namely pMD18T-VP3, PMD18T-VP4, and pMD18T-VP38. Subsequently, the plasmids pMD18T-VP3, pMD18T-VP4, and pMD18T-VP38 and the pFastBac^TM^ HTA were respectively digested by restriction enzyme Not I, Kpn I, Bam I, Xho I, Not I, and Xho I (TaKaRa, Beijing, China). Then, the enzyme-digested fragments were purified and ligated together using T4 DNA ligase (TaKaRa, Beijing, China) at 16 °C for 12 h to generate recombinant plasmid pFastBacHTA^TM^ -VP3, pFastBacHTA^TM^ -VP4, and pFastBacHTA^TM^ -VP38. The target genes were inserted into the pFastBacHTA^TM^ vector under the control of polyhedron promoter (PpH). Next, the ligation mixtures were transformed into *E. coli* DH5α (AngYuBio, Shanghai, China), the DNA was extracted from positive clones and confirmed by sequence analysis, and enzymatic digestion was used for identification. Then, the pFastBacHTA^TM^ -VP3, pFastBacHTA^TM^ -VP4, and pFastBacHTA^TM^ -VP38 plasmids were employed to transform DH10Bac^TM^ competent cells (AngYuBio, Shanghai, China) to produce bacmids. Using the blue-white screening to detect colonies containing recombinant bacmid DNA, they were identified by PCR amplification using the M13 primers. The recombinant bacmids were then isolated and purified using EndoFree Plasmid Maxi kit (QIAGEN, Hilden, Germany) according to the protocol of manufacture.

### 2.3. Acquisition of Recombinant Baculoviruses

To produce the recombinant baculoviruses, Sf9 cells were seeded into six-well plates and transfected with Bacmid-VP3, Bacmid-VP4, and Bacmid-VP38 using Cellfectin II reagent (Gibco, Gaithersburg, MD, USA). In detail, 1.0 × 10^6^ Sf9 cells were incubated into 6-well plates at 27 °C for 24 h before replacing with a ratio of 1:4 Sf9-900 II complete medium and Grace’s Insect Medium (Gibco, Gaithersburg, MD, USA). The mixed medium was supplemented to 1.8 mL, and the cells were incubated for 30 min at room temperature. Then, 1 μg bacmid plasmids and 8 μL Cellfectin II reagent were added to 100 μL of the Grace’s Insect Medium, respectively, and incubated for 30 min at room temperature. Next, the two reagents were gently mixed and incubated for 30 min at room temperature before dispensing onto the Sf9 cells. The whole mixture of approximately 210 μL was added to the cell culture plate and incubated at 28 °C for 4 h before replacing with 2 mL Sf9-900 II complete medium. The viral stock of first passage (shortly named-P1) was harvested from the cell culture until a significant characteristic of viral infection was exhibited and named PFBH-VP3, PFBH-VP4, and PFBH-VP38. Then, amplified baculovirus was stocked until P3 viral stock.

### 2.4. Detection of Recombinant Baculoviruses by Indirect Fluorescence Assay (IFA)

A density of 4 × 10^5^ Sf9 cells were cultured in 24-well plates and infected with the third-generation recombinant baculoviruses. Uninfected Sf9 cells were used as a blank control group. At 4 days of infection, the culture medium was discarded and PBST was used to wash the Sf9 cells three times. Then, the cells were fixed with methanol previously cooled for 10 min at −20 °C. Methanol was discarded and air-dried. The cells were washed three times with PBST for 5 min. Next the cells were incubated with 0.5%Triton for 10 min to permeabilize the cells in a shaking bed at 120 r/min. The solution was discarded and washed three times with PBST for 5 min each. The samples were blocked with 5% skim-milk powder for 30 min at 37 °C, the blocking solution was discarded, and each well was washed three times with PBST as described above. After that, the Sf9 cells were incubated with rabbit anti-VP3, anti-VP4, and anti-VP38 serum (developed in our laboratory) in a dilution of 1:200 for 1.5 h at 37 °C. The cells were washed three times with PBST and incubated with the secondary antibody, a FITC-conjugated goat anti-rabbit antibody (Weiao, Linyi, China), diluted 1:3000 in PBS, for 50 min at 37 °C. Then, PI (Invitrogen, Carlsbad, CA, USA) diluted at 1:10,000 was added to stain cell nuclei for 10 min in the dark at room temperature. All the dilutions were made using PBS. Finally, the cells were cleaned three times with PBST and observed under a fluorescence microscope.

### 2.5. Generation and Purification of GCRV-VLPs

To generate the GCRV-VLPs, Sf9 cells were adjusted to 1.0 × 10^6^ cells/mL and co-infected with PFBH-VP3, PFBH-VP4, and PFBH-VP38 at multiplicity of infection (MOI) of 2:2:1. All infected cultures were incubated for 1 h at 28 °C. After that, Sf9-900 II complete medium was added to the cell cultures and incubated at 28 °C. The Sf9 cells lysates were harvested when an obvious cytopathic effect occurred, which was regarded as the first GCRV-VLPs stock, and amplified GCRV-VLPs were stocked until the third GCRV-VLPs stock. To purify the VLPs, the cell supernatants were subjected to sucrose gradient ultracentrifugation after two repeated freeze-thaw cycles at −20 °C. Cells debris was removed by centrifugation at 5000 r/min for 30 min. Then, the VLPs in the supernatant were pelleted by ultracentrifugation at 37,000 r/min for 2 h at 4 °C. The pellets were resuspended in 500 μL PBS and loaded onto a 20–30–45–60% discontinuous sucrose gradient. The VLPs were collected in different fractions from top to bottom after ultracentrifugation at 37,000 r/min for 1 h at 10 °C and mixed with PBS, respectively. Then, the sample was pelleted by ultracentrifugation at 37,000 r/min for 15 min at 4 °C and resuspended in 100 μL PBS.

### 2.6. Identification and Transmission Electron Microscopy (TEM) Analysis of GCRV-VLPs

The purified VLPs were identified by Western blot analysis. The samples mixed with 5× Loading Buffer were heated at 100 °C for 10 min and centrifuged for 1 min, then separated by 12% SDS-PAGE, and transferred onto NC membranes by electrophoresis. The membranes were then blocked with 5% skim-milk powder in PBS for overnight at 4 °C and then treated with rabbit anti-VP3, anti-VP4, and anti-VP38 sera (developed in our laboratory) diluted at 1:200 for 1.5 h at room temperature. Membranes were rinsed three times with PBST (5 min per wash), followed by incubation for 45 min at room temperature with the goat anti-rabbit secondary antibody in a dilution of 1:5000. Afterward, the membranes were washed three times with PBST (5 min per wash), and the Horseradish Peroxidasechromogenic reagent (Weiao, Linyi, China) was added for development until the bands had been seen clearly. Then, the NC membranes were placed in PBS to terminate the reaction. To investigate whether VLPs were self-assembled correctly, ultrathin sections were analyzed by electron microscopy (EM). Furthermore, negative staining transmission electron microscopy (TEM) was performed to analyze the shape and size of purified GCRV-VLPs. One microliter of purified VLP selected fraction was loaded onto a copper gird and fixed for 5 min, removing the VLPs and loading phosphotungstate on the gird for 10 s. Then grids were air-dried and observed by an electron microscope for TEM analysis.

### 2.7. Immunization of Grass Carp with GCRV-VLPs

The grass carp with body weight of 20 ± 0.5 g and a body length of 10 ± 0.5 cm was purchased from a grass carp farm in Guangzhou, Guangdong, China. They were acclimatized at 28 °C under laboratory conditions for 2 weeks before experimental manipulation, then were maintained in aerated water and fed daily with commercial dry feed pellets (Hello Fish Dry Pellets; CVM Products, Beijing, China). During this observation period the grass carp did not display any clinical signs. Additionally, samples taken from these fish were negative by qRT-PCR for detection of GCRV. The grass carp were divided randomly into five groups (50 fish/group) and injected intraperitoneally for the experiments. For the GCRV-VLPs group, and 30 μg of GCRV-VLPs was used only for the GCRV-VLPs + adjuvant group, 30 μg of GCRV-VLPs was mixed with 100 μL of Montanide IMS 1312 VG adjuvant (Seppic, Paris, France) after vortexing with a final volume of 200 μL. The attenuated vaccine group was immunized with 10 ^7.2^ TICD_50_/mL of 200 μL commercial attenuated vaccine GD1108 strain (Pearl River Fisheries Research Institute, Guangzhou, China).

The PBS group was injected with 200 μL of PBS. The PBS + adjuvant group was injected with PBS mixed with an equal volume of Montanide IMS 1312 VG adjuvant. A boost immunization proceeded 21d later with the same antigens used as the primary immunization. Serum, spleen, and kidney were sampled from five fish in each group three-weeks after immunization and boost immunization and stored at −80 °C.

All animal procedures were conducted according to animal welfare standards and approved by the Ethical Committee for Animal Experiments of Pearl River Fisheries Research Institute, Chinese Academy of Fishery Sciences, China. All animal experiments complied with the guidelines of the Animal Welfare Council of China.

### 2.8. Detection of Serum-Specific IgM Antibody by Enzyme-linked Immunosorbent Assay (ELISA)

The grass carp serum samples were collected to detect specific IgM antibody by indirect ELISA. The virion protein was purified in our laboratory previously. Briefly, 100 μL of purified virion protein was coated onto an ELISA plate as antigen with a concentration of 100 ng/mL and incubated overnight at 4 °C. The next day, the plates were blocked with 5% skim-milk powder in PBS for 2 h at 37 °C. The sera were diluted to 1:200 and were added (100 μL/well) in three replicates and incubated for 1.5 h at 37 °C. Then, 100 μL of HRP-labeled mouse anti-grass carp IgM diluted to 1:10,000 in PBS was added to each well. After an hour of incubation at 37 °C, 90 μL of TMB chromogenic reagent (NCM Biotech, Suzhou, China) was added to each well for 10 min in the dark and then stopped with the TMB stop solution. Optical density (OD) values were measured at a wavelength of 450 nm (OD450) 5 min after the stop solution was added. After each incubated step, the plates were washed with PBST five times (5 min per wash) in a shaking bed at 200 r/min.

### 2.9. Detection of Expression of Immune-Related Genes by Quantitative Real-Time PCR (qRT-PCR)

Total RNA samples were extracted from spleen and kidney of the grass carp from different groups by Trizol Reagen kit (Invitrogen, Carlsbad, CA, USA), according to the manufacturer’s instructions. The cDNAs were synthesized using the PrimeScript^TM^ RT Master Mix (TaKaRa, Beijing, China) and stored at −20 °C. The primers for detecting immune genes are listed in Table 2. With β-actin as the reference gene, expression changes of four immune-related genes containing interferon (IFN), immunoglobulin M(IgM), Toll-like receptor 3 (TLR3), and Toll-like receptor 7 (TLR7) from spleen and kidney were observed to evaluate the immunological effect of the VLPs. The qRT-PCR for the amplification of each target gene was performed in a final volume of 20 μL. The following program was used: one cycle of pre-denaturation at 95 °C for 10 min, then 40 cycles of denaturation at 95 °C for 30 s, and annealing at 60 °C for 40 s. All qRT-PCR reactions were performed in three replicates. The data for each sample were expressed relatively to the expression level of β-actin by using the 2^−ΔΔCt^ method.

### 2.10. GCRV Challenge Tests

On 21 days post-immunization (dpi) after the booster immunization, all groups were challenged intraperitoneally with 10 LD_50_ of 200 μL HuNan1307. The fish were maintained at 28 °C. The mortality and clinical signs such as dark body coloration and hemorrhage of the fines and gill covers were recorded daily for two weeks post-challenge. The relative percent survival (RPS) was calculated as follows: RPS = (1 − the ratio of mortality percent in the immunized group to in the control) × 100%.

### 2.11. Statistical Analysis

The experimental results are shown as means ± SD of the different groups. All statistical analyses were implemented in GraphPad Prism (7.01) and analyzed based on one-way analysis of variance and Tukey tests. *p*-values of <0.05 were considered significant.

## 3. Results

### 3.1. Construction of Recombinant Baculoviruses

**Figure 1:**
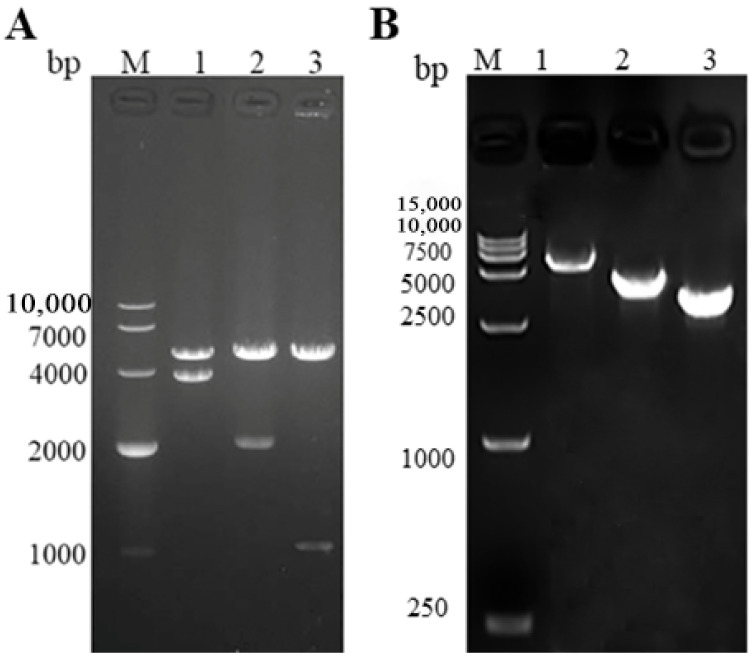
(**A**) Identification of recombinant plasmids by double enzyme digestion. Lane M, DNA marker; lanes 1–3, double-enzyme digestion of pFastBacHTA^TM^ -VP3 (4856 bp 3700 bp), pFastBacHTA^TM^ -VP4 (4856 bp 1953 bp), and pFastBacHTA^TM^ -VP38 (4856 bp 1038 bp). (**B**) Recombinant bacmids were confirmed using PCR analysis with M13. Lane M, DNA marker; lane 1, bacmid-VP3 (6130 bp); lane 2, bacmid-VP4 (4383 bp); lane 3, bacmid-VP38 (3468 bp).

The VP3, VP4, and VP38 proteins encoded by the segments S3, S6, and S10 of the GCRV-HuNan1307 and pFastBacHTA^TM^ were digested to construct the recombinant plasmids pFastBacHTA^TM^ -VP3, pFastBacHTA^TM^ -VP4, and pFastBacHTA^TM^ -VP38. Correct sizes of the recombinant plasmids were identified by dual enzyme digestion, and the results confirmed that the recombinant plasmids were successfully constructed (Figure 1A). The correctly identified recombinant plasmids pFastBacHTA^TM^ -VP3, pFastBacHTA^TM^ -VP4, and pFastBacHTA^TM^ -VP38 were transferred into DH10Bac^TM^ competent cells. The recombinant bacmids were confirmed by colony PCR using M13 forward and reverse primers. The PCR electrophoresis result of the extracted bacmids showed the expected fragment as a clear band of the correct size, without any non-specific amplification (Figure 1B).

### 3.2. Verification of Baculovirus-Expressed GCRV VP3, VP4, and VP38 Proteins

**Figure 2:**
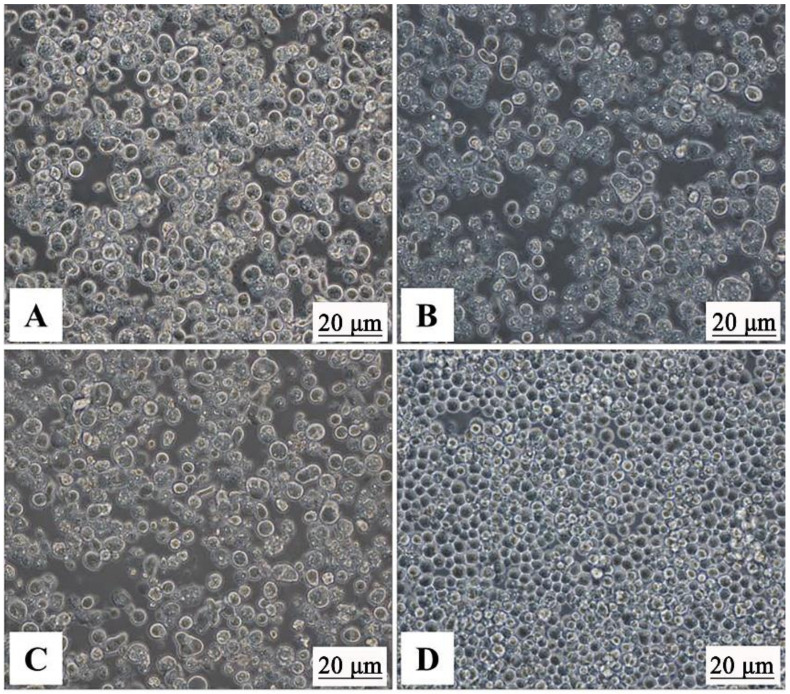
Cytopathic effect of Sf9 insect cells transfected by recombinant bacmids. (**A**) Bacmid-VP3. (**B**) Bacmid-VP4. (**C**) Bacmid-VP38. (**D**) Normal control Sf9 cell.

**Figure 3:**
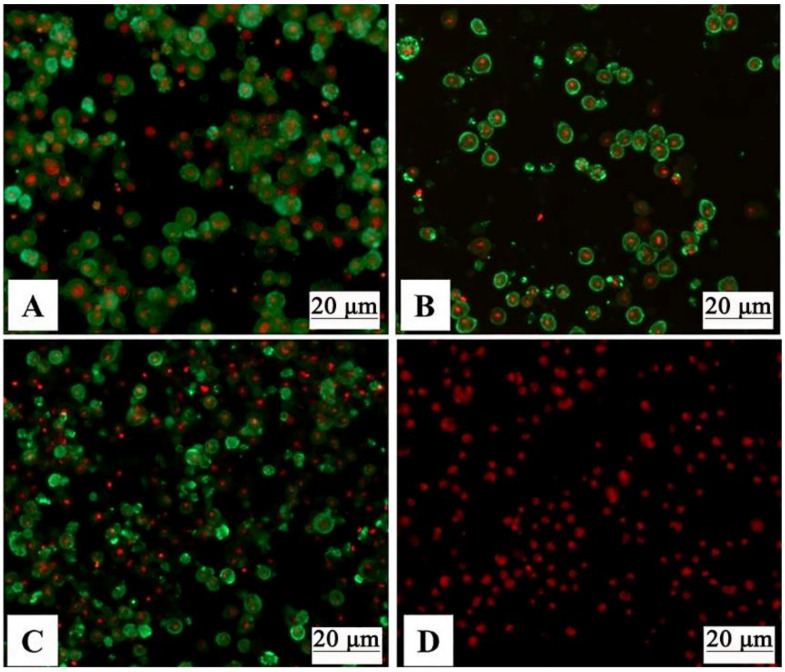
Identification of recombinant baculovirus protein expression by indirect immunofluorescence. (**A**) pFBH-VP3. (**B**) pFBH-VP4. (**C**) pFBH-VP38. (**D**) Normal control Sf9 cell.

To produce the recombinant baculoviruses, Sf9 cells were transfected with Bacmid-VP3, Bacmid-VP4, and Bacmid-VP38. After 72 h of plasmid transfections, the sf9 cells showed increased nucleus and cell volume, and no proliferation was visible (Figure 2A–D). Then, recombinant baculoviruses were harvested from the cell culture, and the third generation of recombinant baculoviruses was identified by IFA. The IFA results showed that sf9 cells infected with PFBH-VP3, PFBH-VP4, or PFBH-VP38 showed a specific green fluorescence signal (Figure 3A–C), but not with non-infected sf9 cells (Figure 3D), which indicated that GCRV VP3, VP4, and VP38 proteins were expressed in infected sf9 cells successfully.

### 3.3. Production and Identification of GCRV-VLPs

**Figure 4:**
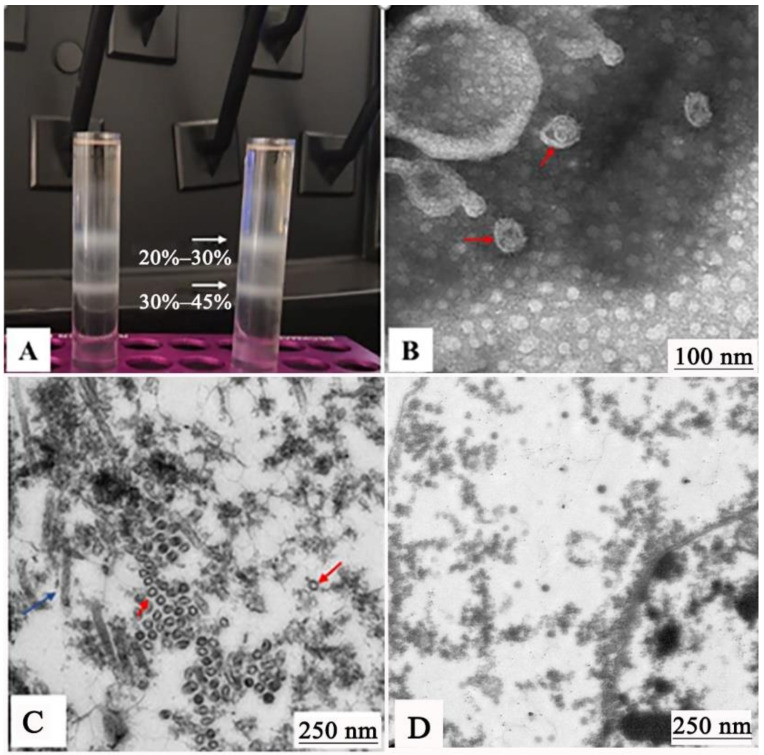
Electron microscopic observation of purified virus-like particles (VLPs) (30,000×) and ultra-thin cell slices (15,000×). (**A**) Sucrose solution with a density of 20–30–45–60%. (**B**) Electron microscopic observation of purified VLPs (30,000×). (**C**) Electron microscopic observation of ultrathin section (15,000×). (**D**) Normal control Sf9 cell.

**Figure 5:**
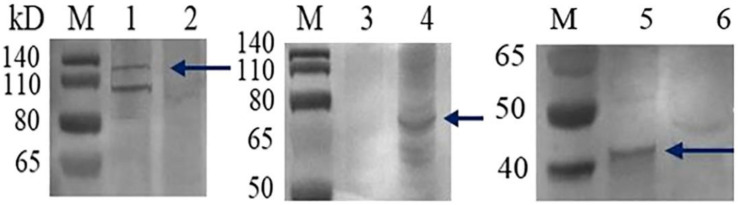
Identification of the VP3, VP4, and VP38 protein components of the GCRV-VLPs by Western blot. M: Low molecular weight protein maker. 1 GCRV-VP3. 2. Normal control Sf9 cell. 3. Normal control Sf9 cell. 4. GCRV-VP4. 5. GCRV-VP38. 6. Normal control Sf9 cell.

To generate the GCRV-VLPs, Sf9 cells were co-infected with PFBH-VP3, PFBH-VP4, and PFBH-VP38. The GCRV-VLPs were harvested from the culture supernatant when an obvious cytopathic effect occurred. To characterize the structure of the GCRV-VLPs, the VLPs were purified by ultracentrifugation and collected at the interface between 20 and 30% as well as 30 and 45% sucrose concentrations (Figure 4A). Then, the purified VLPs were subjected to TEM. The results showed resembled GCRV-VLPs in morphology and shape by retaining the icosahedral symmetry with a diameter between 40 and 60 nm (Figure 4B). The ultrathin section result showed that VLPs were empty particles produced by the recombinant baculoviruses resembling the GCRV in morphology and size, but not from uninfected Sf9 cells (Figure 4C,D). Identification of the VP3, VP4, and VP38 protein components of the GCRV-VLPs was assessed by Western blot. The results showed that corresponding bands of 136 KDa (VP3), 68.34 KDa (VP4), and 38.39 KDa (VP38) were found in the precipitate of the GCRV-VLPs, which were in accordance with the expected size (Figure 5).

### 3.4. Detection of Serum IgM Antibody in Immunized Fish

**Figure 6:**
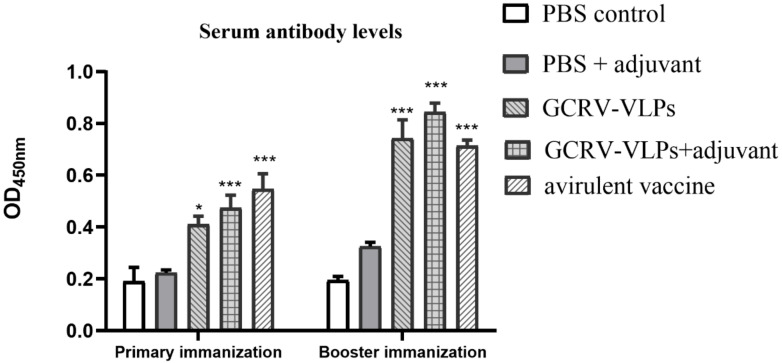
Detection of serum antibodies in grass carp after immunization with VLPs. Purified virion protein was coated onto an ELISA plate as antigen, and the plates were blocked with 5% skim-milk powder in PBS. The sera were added in three replicates. Then, HRP-labeled mouse anti-grass carp IgM was added to each well. After an hour of incubation at 37 °C, TMB chromogenic reagents were added to each well and then stopped with the TMB stop solution. Optical density (OD) values were measured at a wavelength of 450 nm (OD450) 5 min after the stop solution was added. After each incubated step, the plates were washed with PBST five times (5 min per wash). The levels were indicated with OD450 value (* *p* < 0.05, *** *p* < 0.001).

The grass carp serum samples were collected to detect specific IgM antibody by indirect ELISA, and the specific protocol was described above. The ELISA results showed that the antibody titers of the immunized groups increased significantly from 21 dpi of primary immunization compared to the PBS group (*p* < 0.05). On 21 dpi after booster immunization, the antibody titers of the GCRV-VLPs group and GCRV-VLPs + adjuvant group increased significantly compared to the PBS control group, while in the attenuated vaccine group the IgM level was also up-regulated (*p* < 0.001) (Figure 6).

### 3.5. Expression of Immune-Related Genes after the GCRV VLPs-Based Vaccine

**Figure 7:**
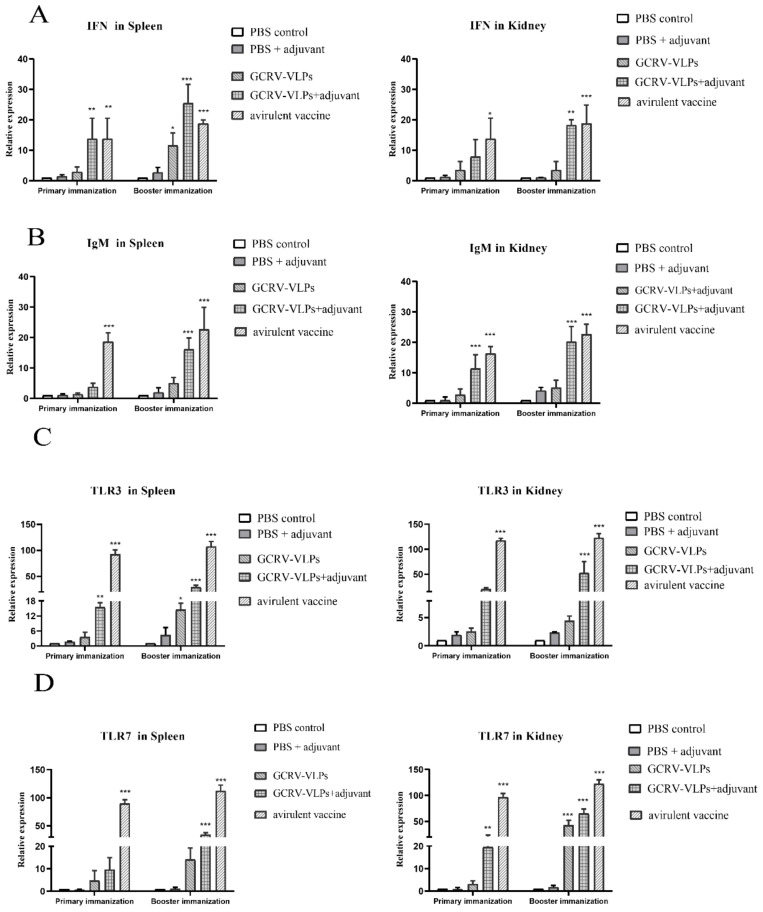
Detection of immune gene expression in immunized grass carp tissues by relative quantitative PCR. (**A**) Relative expression of IFN in spleen and kidney. (**B**) Relative expression of IgM in spleen and kidney. (**C**) Relative expression of TLR-3 in spleen and kidney. (**D**) Relative expression of TLR-7 in spleen and kidney (* *p* < 0.05, ** *p* < 0.01, *** *p* < 0.001).

The spleen and kidney are the largest lymphoid organs in teleosts. The spleen contains antibody-producing cells, which is the major source of immunoglobulins [29]. Therefore, we detected mRNA expression levels of immune-related genes in spleen and kidney tissues by qRT-PCR. In the spleen, the mRNA expression level of IFN in the GCRV-VLPs + adjuvant group increased significantly compared with PBS group after primary immunization (*p* < 0.01). After booster immunization, the expression levels of IFN in every immunized groups were increased higher than that of the PBS group (*p* < 0.05); in addition, the expression level of IFN in the GCRV-VLPs + adjuvant group was above 20-fold higher than that of the PBS group. In the kidney, the expression level of IFN in the GCRV-VLPs + adjuvant group increased significantly compared with PBS group after booster immunization (*p* < 0.01) (Figure 7A). The relative expression levels of IgM in GCRV-VLPs and GCRV-VLPs + adjuvant groups in the spleen were not up-regulated after primary immunization. However, the relative expression level of IgM in the GCRV-VLPs + adjuvant group increased significantly compared with PBS group after booster immunization (*p* < 0.001). In the kidney, the relative expression level of IgM in the GCRV-VLPs + adjuvant group increased significantly compared with PBS group on 21 dpi of both immunization (*p* < 0.01), being approximately 20-fold higher than that of the PBS group (Figure 7B). The expression levels of TLR3 and TLR7 in the GCRV-VLPs + adjuvant group were considerably up-regulated in the spleen and kidney after primary immunization (*p* < 0.01) compared to the PBS group. The expression levels of TLR3 and TLR7 in the GCRV-VLPs group were not up-regulated compared to the PBS group. After booster immunization, the expression levels of TLR3 and TLR7 in spleen and kidney in the GCRV-VLPs + adjuvant group and attenuated vaccine group increased significantly compared to the PBS group (*p* < 0.001) (Figure 7C,D). The results showed that compared with PBS group, the GCRV-VLPs group, GCRV-VLPs + adjuvant group, and attenuated vaccine group increased the mRNA expression of these immune-related genes in the spleen and kidney of grass carp.

### 3.6. Protection of GCRV VLPs-Based Vaccine

**Figure 8:**
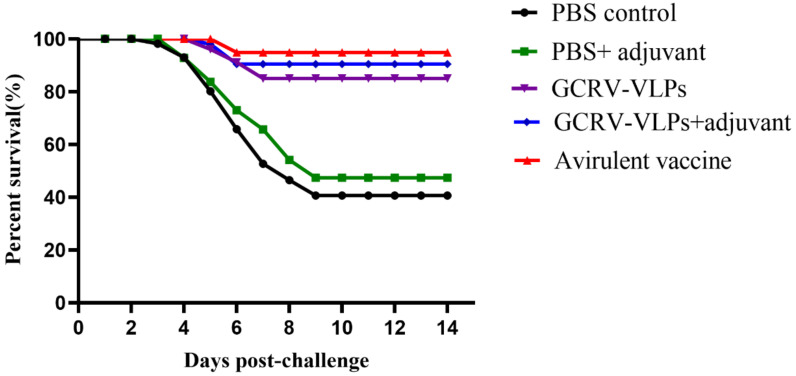
Cumulative survival percentage of the immunized fish after GCRV challenge.

To evaluate the protective effectiveness of the GCRV-VLPs, each group was challenged with a lethal dose of GCRV on 21 dpi after booster immunization. The typical clinical signs of GCHD, including dark body coloration, hemorrhage of the fines and gill covers, began to appear 4 days post-challenge in PBS control group fish. Dead fish samples were confirmed positive of GCRV infection by qRT-PCR. The results showed that the RPS of GCRV-VLPs + adjuvant group was 83.33%, and the RPS of GCRV-VLPs group was 75%; the attenuated vaccine group showed a higher level of protection with 91.67% (Table 3). All the immunized groups had much higher survival rates in comparison to the PBS group (Figure 8).

## 4. Discussion

Infections and diseases induced by GCRV result in extremely high mortality among grass carp, which causes severe economic losses for the grass carp farming industry. Vaccination is a proven efficient way to control virus infection, which makes vaccines a weapon against viral disease. VLP-based vaccines can provide advantages over traditional technologies [30]. The safety and efficacy of VLPs have been proven in various practices. Several VLP-based commercialized vaccines have been produced to protect against some human and livestock viral diseases, such as human papillomavirus (HPV), hepatitis B virus (HBV), and porcine circovirus (PCV) [31]. The first available HBV VLP-based vaccine was licensed in 1986 and resulted in vaccination of more than 300,000 people [32,33]. At present, VLP-based vaccines against fish viral diseases such as infectious pancreatic necrosis virus (IPNV), salmonid alphavirus (SAV), and nervous necrosis virus (NNV) have been developed and tested [14]. The NNV VLPs-based vaccine is the most studied vaccine among VLP-based fish vaccines. Many researchers found that NNV VLP-based vaccines induce a protective immunity [34,35,36]. The current study is the first to develop a GCRV-II VLPs-based vaccine.

In our study, a GCRV-VLPs was developed using a bac-to-bac insect expression system. Recombinant baculoviruses PFBH-VP3, PFBH-VP4, and PFBH-VP38 were generated. Then, sf9 cells were co-infected with recombinant baculoviruses to produce GCRV-VLPs. Purified GCRV-VLPs were similar in morphology to the native virus, as assessed by TEM (Figure 3). Western blot results showed that VP3, VP4, and VP38 proteins assembled GCRV-VLPs efficiently (Figure 4). This suggested that GVRV-VLPs were constructed successfully.

The immunogenicity of the GCRV-VLPs based vaccine was explored through the immunization of grass carp. IgM is the major antibody in teleosts and is regarded as an indicator for the specific immune response [37,38]. In the present study, the grass carp serum samples were collected, and specific IgM antibody was detected by indirect ELISA. The ELISA results showed that the antibody titer induced after immunization with the GCRV-VLPs group and with the GCRV-VLPs + adjuvant group increased significantly at three weeks after immunization and booster immunization compared to the PBS group. The result of the attenuated vaccine group was also an increase after both immunizations compared to PBS group (Figure 6). However, we have not measured the serum antibody levels beyond 21 dpi, so we could not assess the long-term protection afforded by this vaccine. In addition, the mRNA expression of IgM was detected in the spleen and kidney. The relative expression level of the IgM in the GCRV-VLPs + adjuvant group, and also from the attenuated vaccine group in the spleen and kidney, increased significantly compared to the PBS group after booster immunization (Figure 7B). The results demonstrated that GCRV-VLPs can stimulate the production of specific antibodies against the virus in fish.

The IFN system plays an important role in innate immunity as the first line of defense against viral infections [39]. In previous studies, fish IFNs exhibited a powerful capacity to defend against infections with GCRV [40,41]. Meanwhile, fish can exert IFN as an antiviral function through the TLR signaling pathway [42].TLR7 can be involved in immune responses against GCRV in grass carp, and TLR3 is important for resistance to dsRNA analogs and viruses in fish [43]. According to Yuding Fan, the expression levels of TLR3 were significantly up-regulated in gibel carp after injection of Cyprinid Herpes virus-2, which suggested the functions of TLR3 to induce an antiviral immune response [44]. In this study, mRNA expression levels of IFN in GCRV-VLPs + adjuvant group in the spleen and kidney increased significantly compared to PBS group, and the result from GCRV-VLPs + adjuvant group in the spleen was higher than that of the attenuated vaccine group (Figure 7A). The expression levels of TLR3 and TLR7 in the GCRV-VLPs + adjuvant group were increased significantly in the spleen and kidney after primary immunization compared to PBS group, and the attenuated vaccine group was also up-regulated (Figure 7C,D). The results showed that the VLPs increased the mRNA expression of immune-related genes in the spleen and kidney of grass carp, indicating that the GCRV VLPs-based vaccine stimulated the grass carp to generate an antiviral activity and induce innate immune responses.

Additionally, the protective effectiveness of the GCRV VLPs-based vaccine was evaluated the against GCRV challenge. The results showed that the GCRV-VLPs group exhibited RPS of 75% of individual fish, and the GCRV VLPs-based vaccine combined with adjuvant increased the RPS of fish challenged with GCRV to 83.33%. The RPS of the PBS + adjuvant group was 12.5%. The results showed that the adjuvant can improve the protective effectiveness, and the GCRV VLPs-based vaccine can confer effective protection against GCRV infection. Meanwhile, the attenuated vaccine group exhibited RPS of 91.67%. The results showed that the VLPs-based vaccine is not as effective as the attenuated vaccine, while the VLP-based vaccine can overcome the potential risk of reversion to virulence; therefore, safety performance is fine. Although GCRV-HuNan1307 is a representative strain of genotype II that is isolated from grass carp in Hunan province, HuNan1307 could not cause higher mortality rate in our challenge studies, which may partially inflate the survival benefits of the VLP vaccine.

In summary, the present study demonstrated that the GCRV-VLPs based vaccine induced an immune response and conferred effective protection against GCRV infection in grass carp. Therefore, GCRV-VLPs can serve as a potential vaccine with developmental prospects.

## Figures and Tables

**Table 1:** Primer information used in this study.

Gene	Primer	Primer Sequence (5′→3′) ^1^	Restriction Site	Amplicon Size(bp)
S3-VP3	VP3-F VP3-R	5′-ATAGCGGCCGCCCAATGCATCGTCATAACAGA-3′ 5′-ATAGGTACCATGTGCGTGGTCCAGAACGTG-3′	*Not* I *Kpn* I	3700
S6-VP4	VP4-F VP4-R	5′-ATCGGATCCCTGACTTACGGCCACTATCAT-3′ 5′-ATACTCGAGATGCAAAGCGGGGGTCGGTGT-3′	*BamH* I *Xho* I	1953
S10-VP38	VP38-F VP38-R	5′-ATAGCGGCCGCTCAACATGGCGGGTGTGTCTC-3′ 5′-ATACTCGAGGTACCGTTATCGCCGACCACA-3′	*Not* I *Xho* I	1038
M13	M13-F M13-R	5′-GTTTTCCCAGTCACGAC-3′ 5′-CAGGAAACAGCTATGAC-3′		2430 bp + the inset

^1^ The underlined nucleotides indicate restriction sites.

**Table 2:** Primer sequences for immune factors detection.

Gene	Primer Sequence (5′→3′)	GenBank Accession
β-actin-F	5′-GGATGATGAAATTGCCGCACTGG-3′	M25013.1
β-actin-R	5′-ACCGACCATGACGCCCTGATGT-3′	
IFN-F	5′-GACACATACAGTAGGATATTCACTCGC-3′	DQ357216.1
IFN-R	5′-TTGCCTGGGAAGTAGTTTTCTTG-3′	
TLR3-F	5′-GAGAACAATCGTGACTCCCTGA-3′	DQ885910.1
TLR3-R	5′-CCAGTAGAGAACACAGCGAGGT-3′	
IgM-F	5′-TCTACCTCCAACTCCACCACC-3′	DQ417927.1
IgM-R	5′-TGTTTATTGTATTTGCCACCTGAT-3′	
TLR7-F	5′-GAGCATACAGTTGAGTAAACGCAC-3′	AB553573.1
TLR7-R	5′-TCTCCAAGAATATCAGGACGATAA-3	

**Table 3:** Mortality rate and relative percentage survival (RPS) of fish challenged with GCRV.

Project	Mortality Rate (%)	Survival Rate (%)	RPS ^2^ (%)
PBS control	60	40.00	--
PBS + adjuvant	52.5	47.50	12.50
Attenuated vaccine	5.00	95.00	91.67
GCRV-VLPs	15.00	85.00	75.00
GCRV-VLPs + adjuvant	10.00	90.00	83.33

^2^ RPS = (1 − the ratio of mortality percent in the immunized group to in the control) × 100%.
